# Baseline gene expression profiling determines long-term benefit to programmed cell death protein 1 axis blockade

**DOI:** 10.1038/s41698-022-00330-3

**Published:** 2022-12-15

**Authors:** Ioannis A. Vathiotis, Leonidas Salichos, Sandra Martinez-Morilla, Niki Gavrielatou, Thazin Nwe Aung, Saba Shafi, Pok Fai Wong, Shlomit Jessel, Harriet M. Kluger, Konstantinos N. Syrigos, Sarah Warren, Mark Gerstein, David L. Rimm

**Affiliations:** 1 Department of Pathology, Yale School of Medicine, New Haven, CT USA; 2 Yale Cancer Center, Yale School of Medicine, New Haven, CT USA; 3 Program in Computational Biology and Bioinformatics, Yale University, New Haven, CT USA; 4 Department of Molecular Biophysics and Biochemistry, Yale University, New Haven, CT USA; 5 Department of Biological and Chemical Sciences, New York Institute of Technology, New York, USA; 6 Section of Medical Oncology, Department of Internal Medicine, Yale School of Medicine, New Haven, CT USA; 7 Department of Internal Medicine, National and Kapodistrian University of Athens School of Medicine, Athens, Greece; 8 NanoString Technologies, Seattle, WA USA; 9 Department of Computer Science, Yale University, New Haven, CT USA; 10 Department of Statistics and Data Science, Yale University, New Haven, CT USA

**Keywords:** Predictive markers, Melanoma

## Abstract

Treatment with immune checkpoint inhibitors has altered the course of malignant melanoma, with approximately half of the patients with advanced disease surviving for more than 5 years after diagnosis. Currently, there are no biomarker methods for predicting outcome from immunotherapy. Here, we obtained transcriptomic information from a total of 105 baseline tumor samples comprising two cohorts of patients with advanced melanoma treated with programmed cell death protein 1 (PD-1)-based immunotherapies. Gene expression profiles were correlated with progression-free survival (PFS) within consecutive clinical benefit intervals (i.e., 6, 12, 18, and 24 months). Elastic net binomial regression models with cross validation were utilized to compare the predictive value of distinct genes across time. Lasso regression was used to generate a signature predicting long-term benefit (LTB), defined as patients who remain alive and free of disease progression at 24 months post treatment initiation. We show that baseline gene expression profiles were consistently able to predict long-term immunotherapy outcomes with high accuracy. The predictive value of different genes fluctuated across consecutive clinical benefit intervals, with a distinct set of genes defining benefit at 24 months compared to earlier outcomes. A 12-gene signature was able to predict LTB following anti-PD-1 therapy with an area under the curve (AUC) equal to 0.92 and 0.74 in the training and validation set, respectively. Evaluation of LTB, via a unique signature may complement objective response classification and characterize the logistics of sustained antitumor immune responses.

## Introduction

With incidence rates rising over the past 40 years, melanoma is projected to cause about 100,000 new cases and 8000 deaths in the United States in 2022^1–3^. Development of monoclonal antibodies targeting the cytotoxic T-lymphocyte antigen-4 (CTLA-4; ipilimumab, approved by the FDA in 2011) and programmed cell death protein 1 (PD-1; nivolumab, pembrolizumab, approved by the FDA in 2014) has dramatically improved outcomes for patients with advanced disease; five-year survival rates have climbed to 52% for previously untreated patients receiving the combination of ipilimumab and nivolumab and 41–44% for those receiving anti-PD-1 monotherapy^4,5^. More importantly, unlike classic chemotherapy or targeted therapies, pivotal clinical trials for immune checkpoint inhibitors (ICIs) have documented a plateau of durable responses apparent at the tail of the survival curves. In the real world, a large fraction of long-term survivors may be off-treatment and have no active disease, having required only immune checkpoint inhibition and at some point, local therapy for residual or oligometastatic disease^6^.

Currently, there are no FDA-approved biomarkers predictive of response to ICIs in patients with melanoma. Baseline assessment of programmed death-ligand 1 (PD-L1) expression by immunohistochemistry, tumor infiltration by CD8-positive T cells, evidence of an inflamed tumor microenvironment (TME) driven by active interferon gamma (IFNγ) signaling detected by gene expression profiling, or high tumor mutational burden (TMB) have shown limited predictive value when tested prospectively or have been challenging to implement in the clinic^7–15^. Moreover, the evaluation of objective response rate by standard radiographic Response Evaluation Criteria in Solid Tumors (RECIST) 1.1 systematically fails to capture certain patterns of response to immunotherapy and may underestimate therapeutic benefit from ICIs^16,17^. For patients who improve with immune checkpoint inhibition, categorical definition of benefit is critical. Determination of the optimal duration of treatment is also needed, with implications to treatment-related toxicity, quality of life and health economics; several studies have suggested that a limited rather than continued course of treatment may be sufficient to produce profound responses^18^. Conversely, for those who develop primary or acquired resistance to ICIs, implementation of novel approaches, including new immunotherapy combinations, through clinical trials may prove valuable.

Here, we sought to determine whether long-term benefit (LTB) to immunotherapy represents a biologically distinct entity compared with objective response or short-term outcomes. To do so, we acquired baseline transcriptomic information from two cohorts of patients with melanoma treated with PD-1-based immunotherapies. Instead of objective response classification per RECIST 1.1, we utilized progression-free survival (PFS) exclusively to separate patients who benefit from immune therapy, from those who do not. We provide proof of concept that different gene expression profiles are associated with outcome at subsequent timepoints post treatment initiation. We further develop and validate a signature that can accurately predict the benefit from immunotherapy at 24 months, characterizing a subgroup of long-term survivors that reside in the tail of the survival curves. Such patients are essentially candidates for functional cure and should probably be managed in a different manner from those who show a response by RECIST 1.1.

## Results

### Cohort characteristics

**Fig. 1:**
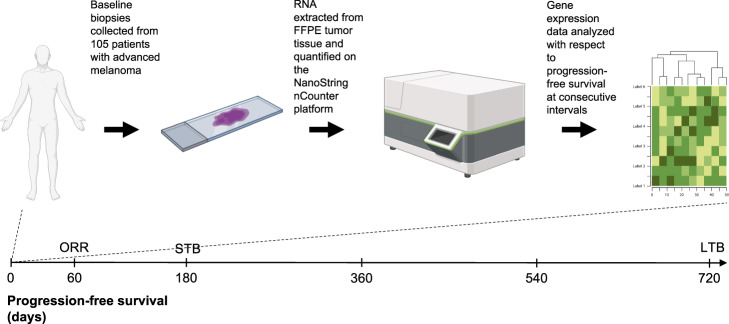
Study workflow. Development of gene signatures that predict LTB to programmed cell death protein 1-based immunotherapies in patients with melanoma. Created with BioRender.com. FFPE formalin-fixed paraffin-embedded, ORR objective response rate, STB short-term benefit, LTB long-term benefit.

A total of 105 patients were included in the analysis (Fig. 1). Median age of study participants was 63 years, ranging from 16 to 88 years. Sixty patients were males and 45 were females. Activating *BRAF* and *NRAS* mutations were present in 32 (31%) and 18 (17%), respectively. All patients received PD-1-based immunotherapies in the advanced setting. Fifty-eight patients (55%) received anti-PD-1 monotherapy, including 32 treated with pembrolizumab and 26 treated with nivolumab, and 47 patients (45%) received combination immunotherapy with anti-CTLA-4 plus anti-PD-1 (ipilimumab plus nivolumab). It should be noted that 22 patients (21%) had received ipilimumab monotherapy in a previous line of treatment. ORR was 43%. Disease progression at data cutoff was documented in 67 patients (64%). Median PFS for the study cohort was 7.4 months. STB was observed in 42 patients (40%); LTB, calculated at 24 months, was observed in 22 patients (21%). Forty-three patients (41%) survived until data cutoff and median overall survival (OS) for the study cohort was 20.1 months. Detailed cohort characteristics can be seen in Table 1.

**Table 1:** Patient characteristics.

Characteristic	Discovery cohort (2011–2017)	Validation cohort (2017–2020)	Total
	*N* (%)	*N* (%)	*N* (%)
Overall	59 (100)	46 (100)	105 (100)
Age			
Median (range)	62 (16–88)	66 (31–88)	63 (16–88)
Sex			
Male	33 (56)	27 (59)	60 (57)
Female	26 (44)	19 (41)	45 (43)
Stage			
II	0 (0)	2 (4)	2 (2)
III	1 (2)	5 (11)	6 (6)
IV	58 (98)	39 (85)	97 (92)
Mutation status			
BRAF	18 (31)	14 (30)	32 (31)
NRAS	8 (14)	10 (22)	18 (17)
None	32 (54)	20 (43)	52 (50)
Prior immune checkpoint blockade			
Yes	16 (27)	6 (13)	22 (21)
No	43 (73)	40 (87)	83 (79)
Treatment			
Pembrolizumab	23 (39)	9 (20)	32 (31)
Nivolumab	11 (19)	14 (30)	25 (24)
Ipilimumab plus nivolumab	25 (42)	23 (50)	48 (46)
Best overall response			
Complete response	10 (17)	11 (24)	21 (20)
Partial response	16 (27)	8 (17)	24 (23)
Stable disease	17 (29)	8 (17)	25 (24)
Progressive disease	16 (27)	18 (39)	34 (32)
Long-term benefit			
Yes	13 (22)	9 (20)	22 (21)
No	37 (63)	27 (59)	64 (61)

*CR* complete response, *PR* partial response, *SD* stable disease, *PD* progressive disease.

### Baseline gene expression profiles are consistently able to predict LTB to PD-1 axis blockade

**Fig. 2:**
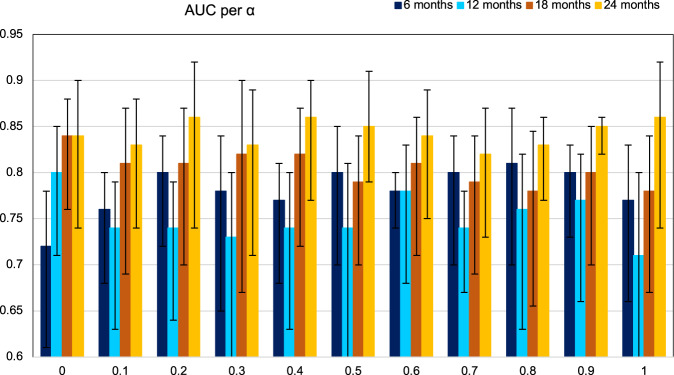
Baseline gene expression profiling is accurate at predicting long-term immunotherapy outcomes. Comparison of elastic net binomial models designed for the prediction of PFS at consecutive 6-month intervals across α values ranging from 0 to 1; α equals “0” for ridge and “1” for lasso regression. Models designed for the prediction of PFS at 24 months perform consistently better in comparison with models designed for the prediction of PFS at 6, 12, and 18 months. The error bars correspond to the 95% confidence intervals. PFS progression-free survival.

To predict clinical benefit from ICIs at different timepoints (i.e., 6, 12, 18, and 24 months post treatment initiation) based on gene expression, we first tested a series of elastic net binomial models with α ∊ [0,1] with increments of 0.1 (α equals ‘0 ‘for ridge and ‘1’ for lasso regression; Fig. 2). In general, α = 0.8 appeared to provide consistent AUC values at all four timepoints as well as highest performance for the model predicting STB. Alpha values close to ‘1’ approach a lasso regression model, which reduces the number of genes to avoid overfitting. Overall, our models showed the highest accuracy for predicting clinical benefit at 24 months (LTB), with AUC values consistently over 0.8. These results suggested that different genes account for the prediction of PFS across time.

### The predictive value of distinct genes fluctuates across time

**Fig. 3:**
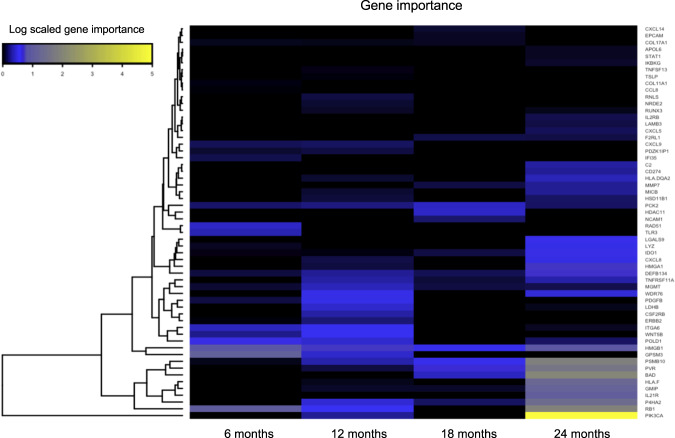
Variability in the predictive value of genes across consecutive clinical benefit intervals. Heatmap of gene importance across time. The predictive value of certain genes varies in respect of time. A distinct set of genes appears to “switch on” at 24 months post treatment initiation.

To examine the potential variability in the predictive value of distinct genes across consecutive clinical benefit intervals, we trained four different elastic net binomial models with α = 0.8, one for each of the four clinical benefit subcategories. Then, for every model we calculated gene importance using variant important scores. Finally, we compared each gene’s importance across all four clinical benefit intervals by fitting a linear curve and testing for positive or negative association. Overall, our results indicated the inclusion or exclusion of different genes as PFS increases (Fig. 3). Indicatively, *PVR* and *MMP7* showed the highest correlation coefficient (*R* = 0.94) with benefit at 24 months, while *GPSM3* showed the lowest (*R* = −0.93), indicating minor importance in the prediction of benefit at 24 months. Despite the small number of timepoints sampled, *PVR* and *MMP7* had *p*-values of 0.05 and 0.06, respectively. Similarly, genes such as *STAT1* or *APOL6*, had very large positive linear coefficients (based on linear fit models) indicating a potential inclusion, while genes like *CCL8* and *COL11A1* the most negative.

### A 12-gene signature predicts LTB to PD-1 axis blockade in patients with melanoma

**Fig. 4:**
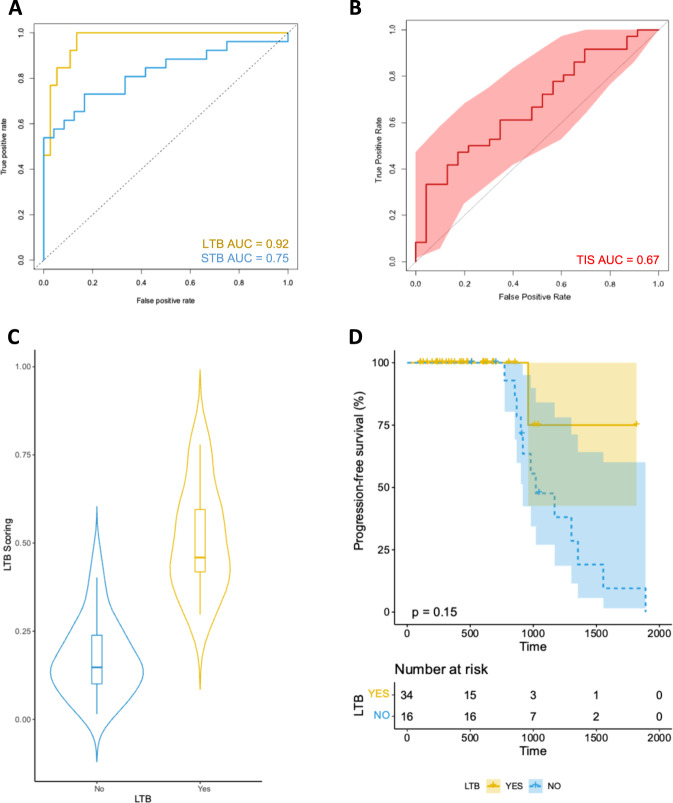
Development of the 12-gene signature that predicts LTB from programmed cell death protein 1-based immunotherapies in patients with advanced or metastatic melanoma. **A** Receiver operating characteristic curves for LTB and STB signatures in the discovery set. **B** Receiver operating characteristic curves for the TIS signature in the discovery set. **C** Violin plot showing the distribution of scores according to LTB outcome in the discovery set. **D** Kaplan–Meier curve for PFS for patients ranked high according to the 12-gene LTB signature score in the discovery set. For the training set the low subgroup has a minimum score of 0.0007, 1st quantile of 0.045, median of 0.11, mean of 0.18, 3rd quartile of 0.28 and maximum score of 0.54; the high subgroup has a minimum score of 0.25, 1st quartile of 0.41, median of 0.53, mean of 0.53, 3rd quartile of 0.63 and maximum value of 0.89. The error bars correspond to the 95% confidence intervals. LTB long-term benefit, STB short-term benefit, AUC area under the curve, PFS progression-free survival.

To identify a final prediction model with a reduced number of genes that can potentially provide us with a future gene signature for LTB, we utilized the discovery cohort (*n* = 59) to apply a Lasso binomial regression in our set of 58 genes (*α* = 1; Fig. 4A). We optimized parameter lambda (*λ* = 0.0207) using cross validation and AUC from 1000 runs. Overall, our model identified coefficients for 12 genes (*CXCL8*, *DEFB134*, *ERBB2*, *HLA.DQA2*, *IDO1*, *INHBA*, *MMP7*, *MYD88*, *NRDE2*, *P4HA2*, *PIK3CA*, and *PSMB10*; Supplementary Table 2), with a maximum AUC equal to 0.92 (95% confidence intervals [CI], 0.85–0.99; Fig. 4A). Notably, our LTB signature performed significantly better than the already published Tumor Inflammation Signature (TIS; AUC, 0.67; 95% CI, 0.54–0.82; Fig. 4B) in the discovery cohort^10,19,20^. Moreover, our optimized model for the prediction of STB had an AUC of 0.75 (95% CI, 0.63–0.87); of the genes included in the LTB model, only *CXCL8* and *HLA.DQA2* accounted for the prediction of STB. Our LTB model had an accuracy of 0.86, sensitivity of 1, specificity of 0.46, PPV of 0.84, and NPV of 1. The p-value for accuracy versus non-informative rate (NIR) was 0.03. In addition, based on the Youden’s index, we identified the optimal cutoff for our LTB scoring (LTB score = 0.32; Fig. 4C, D). Our LTB model performed poorly in the TCGA melanoma dataset (patients not treated with immunotherapy), suggesting lack of prognostic value and immunotherapy specificity (Supplementary Fig. 1).

**Fig. 5:**
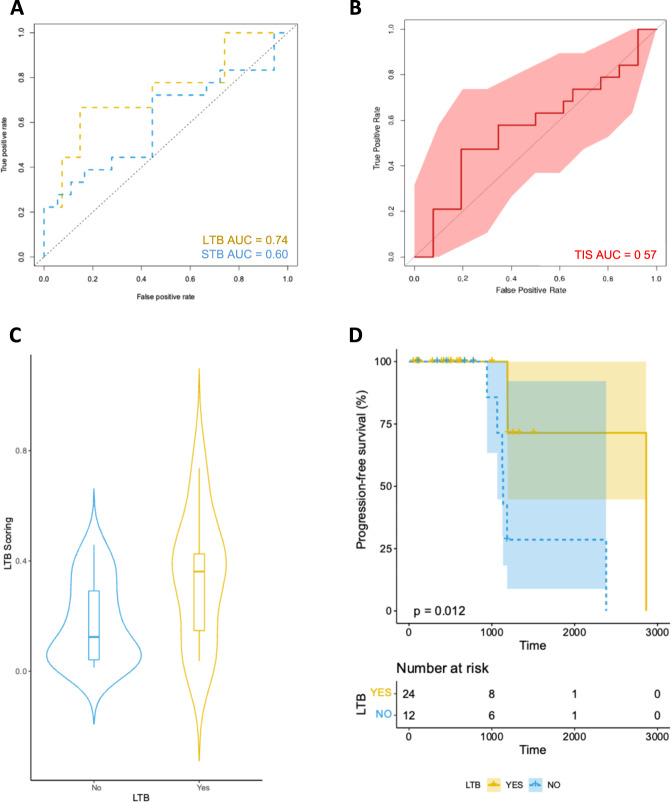
Validation of the 12-gene signature that predicts LTB from programmed cell death protein 1-based immunotherapies in patients with advanced or metastatic melanoma. **A** Receiver operating characteristic curves for LTB and STB signatures in the validation set. **B** Receiver operating characteristic curves for the TIS signature in the validation set. **C** Violin plot showing the distribution of scores according to LTB outcome in the validation set. **D** Kaplan–Meier curve for PFS for patients ranked high according to the 12-gene LTB signature score in the validation set. For the validation set the low subgroup has a minimum score of 0.0001396, 1st quartile of 0.018, median of 0.09, mean of 0.12, 3rd quartile of 0.18 and maximum score of 0.39; the high subgroup has a minimum score of 0.1880, 1st quartile of 0.32, median of 0.7, mean of 0.57, 3rd quartile of 0.74 and maximum score of 0.84. The error bars correspond to the 95% confidence intervals. LTB long-term benefit, STB short-term benefit, AUC area under the curve, PFS progression-free survival.

The 12-gene signature was validated with a second independent immunotherapy-treated melanoma cohort (validation cohort), where it predicted LTB with an AUC of 0.74 (95% CI, 0.52–0.95; Fig. 5A). On the same cohort, the TIS and STB model exhibited an AUC of 0.57 (95% CI, 0.39–0.75) and 0.60 (95% CI, 0.42–0.80), respectively (Fig. 5B). Using the optimal cutoff, patients with high LTB score had significantly prolonged PFS compared with patients with low LTB score in the validation cohort (log rank test; *p* = 0.012; Fig. 5C, D). Furthermore, our set of 11 genes (excluding *HLA.DQA2*) was highly predictive of LTB in a third external validation cohort (Gide et al.) with an AUC of 0.87 (95% CI, 0.82–0.92; Supplementary Fig. 2).

## Discussion

In this proof-of-concept study, we used a simple, sensitive, and quantitative approach, compatible with tissue-limiting FFPE tumor specimens routinely obtained in the clinic, to capture transcriptomic information portraying the complex interplay between the tumor and the TME. We observed that baseline immune gene expression profiling was more accurate at predicting long-term immunotherapy outcomes (at 24 months post treatment initiation) compared with short-term (<24 months post treatment initiation). Next, we documented a switch in the predictive value of single genes when evaluated at consecutive timepoints; while certain genes are associated with response early in the course of treatment, others appear to dominate for patients with LTB at 24 months. Finally, through a rigorous multistep process, we developed a 12-gene signature that predicted LTB to PD-1-based immunotherapies in patients with melanoma.

The role of TIS in predicting objective response to immunotherapy has been established previously. In their study, Cristescu et al. used two clinical trial cohorts (KEYNOTE-001, KEYNOTE-006) comprising a total of 89 patients with advanced melanoma that were treated with pembrolizumab^20^. They found that TIS was able to predict objective response with an AUC of 0.64. In our study, we used 2 real-world cohorts comprising a total of 105 patients with advanced melanoma that were treated with either of the three FDA-approved regimens for advanced disease (nivolumab, pembrolizumab, nivolumab plus ipilimumab). We showed that TIS was able to predict objective response with an AUC of 0.67 in the discovery cohort (*n* = 59) and an AUC of 0.57 in the validation cohort (*n* = 46). Despite any differences in treatment regimens, the combined AUC of both our cohorts would be extremely close to that of Cristescu et al. and this is in support of our findings.

To evaluate benefit following treatment with ICIs we show that conventional objective response classification based on radiographic RECIST 1.1 may be less valuable in thinking about patient gain. Bidimensional tumor shrinkage typically characterizes tumor cell death as a result of cytotoxic chemotherapy or targeted therapies^21^. However, classic RECIST 1.1 do not capture atypical patterns of response, such as mixed response or pseudoprogression, occasionally seen in patients receiving ICIs^17^. In addition, radiographic assessment of response does not always correlate with time to progression^6^. Although complete eradication of tumor lesions may predict prolonged PFS, documentation of a partial response or even stable disease does not preclude sustained antitumor responses^22^. We alternately assessed treatment benefit as a binary variable (i.e., yes, no) for patients who remained progression-free over successive 6-month intervals. While classic chemotherapy might perform comparably, or even better than immunotherapy during the first six months of treatment, immune therapies produce a plateau or flattening at the tail of the survival curves, conferring durable survival benefit in a subset of patients^23,24^. In fact, matching-adjusted indirect comparisons of dual immune checkpoint blockade with ipilimumab and nivolumab versus combined BRAF and MEK inhibition in patients with melanoma show that tentative benefits from immunotherapy emerge after the first 12 months of treatment^25^. In CheckMate 067, PFS curves begin to flatten after ~24 months, irrespective of immunotherapy agent administered; unconfined by the effect of subsequent anticancer therapies, the flattening of the PFS curve at 24 months appears quite stable thereafter representing a useful surrogate for long-term immunotherapy outcomes (i.e., PFS at five years post treatment initiation, OS)^4,26–28^.

The fact that the predictive value of certain genes fluctuates based on the timepoint at which PFS is assessed is the most notable finding from the study. Our data indicate a potential positive switch for *CD274* at 24 months post treatment initiation. Although PD-L1 positivity by IHC has limited predictive value for objective response classification in patients with melanoma, elevated levels of *CD274* mRNA at baseline might explain, to some degree, durable responses to PD-1-based immunotherapies^29^.

Multigene signatures represent an effective approach to characterize the dynamics between the tumor and the TME. In fact, they can simultaneously outline and quantify multiple aspects of the latter that are linked with response to therapy. Using a lasso regression model and stepwise cross validation to minimize the risk of overfitting, we identified a set of genes that can accurately select patients who will experience stable tumor regression. Our final 12-gene LTB signature encompassed two major components of the tumor/TME interaction. The first component pertains to the hallmarks of cancer including relentless proliferation, evasion of growth suppression, resistance to apoptosis, and activation of invasion and metastasis. To this regard, increased baseline *PIK3CA* expression showed a high-positive association with LTB. Preclinical data support that PD-L1 expression levels are not influenced by oncogenic events in the phosphatidylinositol 3-kinase (PI3K) signaling pathway^30^. However, increased PI3K/AKT pathway activation, as documented in patients with melanoma harboring *BRAF V600K* mutations, correlated with high tumor mutational load and improved immunotherapy outcomes^31^. The second component appears to relate to the immune response towards the primary tumor. Current literature has linked objective response to immunotherapy with increased local IFNγ production^10^. Our findings clearly suggest a role for preexisting IFNγ signaling as well as enhanced antigen presentation in LTB from PD-1-based immunotherapies. As a matter of fact, *IDO1* and *PSMB10* that have been implicated in the prediction of objective response by Ayers et al. also take part in the prediction of LTB in our model; interestingly, both genes appear to gain importance over time.

Previous efforts from our group led to the generation of a mixed RNA and protein model (YMMM) for the prediction of best overall response (BOR) to immune checkpoint inhibition in patients with melanoma^32^. Notably, only *NRDE2* contributed to the prediction of both BOR and LTB. Nuclear RNAi-defective 2 (NRDE2) is an evolutionarily conserved protein involved in RNA splicing; *NRDE2* is responsible for suppressing intron retention with a tropism for pre-mRNAs with short, weak, GC-rich introns^33,34^. In addition, loss of *NRDE2* results in severe genomic instability with accumulation of double-strand breaks^35^. In our models, *NRDE2* demonstrated an inverse correlation with outcome. Hence *NRDE2*-dependent genomic events might lead to the aggregation of neoantigens rendering melanoma tumors susceptible to PD-1-based immunotherapies.

From a clinical standpoint, baseline computation of LTB, with further validation, could be a valuable complement to current companion diagnostic tests that focus on objective response classification. It incorporates follow-up information spanning a period of two years and enables simultaneous evaluation of immunotherapy outcomes at a second time point. Thus, assessment of LTB could increase confidence in the point estimate of objective response and, with a maximum sensitivity, identify false negative cases. Also, it may mark candidates for “functional cure”, with implications to treatment discontinuation, and others in need of more intensive or alternative treatment approaches^36^. Finally, evaluation of LTB may provide useful insights into the evolving interactions between cancer cells and the host immune system that allow tumors evade recognition according to the immunoediting theory^37^.

This study is subject to several limitations. First, the study cohorts were collected retrospectively. Second, patients received either single agent or combination immunotherapy. The fact that the development of the 12-gene signature was based on previous selection of 770 genes contained in the nCounter PanCancer IO 360™ Panel represents an additional limitation of our study compared to an unbiased examination of the transcriptome. The prognostic value of the genes that comprise the 12-gene signature requires prospective evaluation in randomized trials (versus a “no treatment” arm), which would be challenging or impossible from an ethical perspective. Finally, this is a proof-of-concept study and further analyses are pending to standardize the 12-gene LTB signature for different normalization methods used in RNA sequencing versus NanoString-obtained datasets and thus, ensure wide applicability across centers.

In conclusion, assessment of LTB allows for the evaluation of benefit at 24 months and provides deeper understanding of the biology relating to treatment with ICIs. Added to the assessment of objective response, it may evolve into a biomarker approach that is tailor-made to immune therapies, representing a paradigm shift in biomarker design.

## Methods

### Patient cohorts

The study cohorts are retrospective collections of melanoma patients with available tissue, treated with PD-1-based immunotherapies in the advanced setting from 2011 to 2017 (discovery cohort) and from 2017 to 2020 (validation cohort) at Yale Cancer Center (New Haven, CT). Patients with uveal melanoma were excluded. Pretreatment formalin-fixed, paraffin-embedded (FFPE) specimens from Yale Pathology archives were reviewed by a board-certified pathologist. Clinicopathological data were collected from clinical records and pathology reports; the data cutoff date was September 1, 2020. RECIST 1.1 were used to classify best overall response as complete response (CR), partial response (PR), stable disease (SD), or progressive disease (PD), and to determine the objective response rate (ORR)^16^. PFS was utilized to generate successive, 6-month clinical benefit intervals; short-term benefit (STB) was defined for patients who were alive and free of disease progression within 6 months from treatment initiation and long-term benefit (LTB) for those who were alive and free of disease progression within 24 months from treatment initiation. Patients whose follow-up was shorter than the prespecified intervals were excluded from the analysis. All patients provided written informed consent. The study was approved by the Yale Human Investigation Committee protocol #9505008219 and conducted in accordance with the Declaration of Helsinki.

Clinical data were also downloaded from the Genomic Data Commons (GDC) data portal from The Cancer Genome Atlas (TCGA) Research Network: https://cancergenome.nih.gov. Overall survival (OS) annotation was provided by TCGA for 470 patients with primary melanoma not treated with immunotherapy (TCGA-SKCM).

Finally, publicly available RNA sequencing information from pretreatment samples of patients with advanced melanoma treated with immune checkpoint inhibitors were downloaded to obtain external validation of our LTB gene signature^38^. Given that this dataset used TPM normalization for gene expression (rather than gene count normalization based on a set of core genes from the NanoString nCounter platform), and that *HLA.DQA2* gene was absent, we reworked our model’s weights to test the predictive value of the remaining 11 genes for LTB.

### Quality assessment of FFPE tissue specimens

Similar to previous studies, one cut section from each tissue block was routinely stained with hematoxylin and eosin and coverslipped for assessment of sample quality. Each hematoxylin and eosin-stained slide was reviewed by a board-certified pathologist to evaluate the adequacy of tumor cells, the quality of tissue preservation, and whether significant artifacts relating to fixation, processing, or prefixation tissue handling were present. Specimens containing no or minimal tumor tissue were excluded from the analysis^10^.

### RNA isolation and gene expression analysis

Total RNA was isolated from 5 μm-thick pretreatment FFPE sections of tumors fixed on positively charged slides using the High Pure FFPET RNA Isolation Kit (Roche) following the manufacturer’s protocols. RNA was quantified using the NanoDrop ND1000 spectrophotometer (Thermo Fisher Scientific). Gene expression analysis was conducted on the NanoString nCounter platform (NanoString Technologies). The nCounter PanCancer IO 360™ Panel containing 770 genes related to the tumor, its microenvironment and the antitumor immune response was used. Per sample, 250 ng of total RNA in a final volume of 5 μl was mixed with a 3′ biotinylated capture probe and a 5′ reporter probe and tagged with a fluorescent barcode from the custom gene expression code set. Probes and target transcripts were hybridized at 67 °C for 16–24 h per the manufacturer’s recommendations. Hybridized samples were run on the NanoString nCounter preparation station using the high-sensitivity protocol, in which excess capture and reporter probes were removed and transcript-specific ternary complexes were immobilized on a streptavidin-coated cartridge. The cartridge was scanned at maximum scan resolution on the nCounter Digital Analyzer^10^.

### Normalization

Raw data counts were normalized using the geomean of 10 housekeeping genes included in the nCounter PanCancer IO 360™ Panel and each gene was adjusted based on the average of 2 panel standards across all data. Then, the housekeeper- and panel standard-normalized data were log2 transformed.

### Statistical analysis

Our initial dataset consisted of 770 genes. To reduce the initial number of genes, we first implemented a correlation analysis between each gene’s expression and clinical benefit at subsequent timepoints (6, 12, 18, and 24 months post treatment initiation) in our cohort. For each gene correlation, we collected the resulting non-adjusted *p*-value. Then, we selected those with a non-adjusted *p*-value of < 0.05 for each timepoint and pooled them together. This resulted in a final list of 58 genes, containing those associated with clinical benefit in at least one timepoint (Supplementary Table 1). Elastic net binomial regression models were inferred using (*glmnet*) package. Cross validation was performed using (*cv.glmnet*). For every model we optimized the lambda parameter (λ; nlamda = 1000) using an 8-fold cross validation and maximum AUC. To determine the best AUC across different alpha values (*α* from 0 to 1 with 0.1 increments) we used 100 replicates. Across all α values, we selected *α* = 0.8 for our elastic net models since all four models exhibited solid performance (AUC > 0.75), and the performance of the STB model (PFS at 6 months) was highest. To determine model coefficients for *α* = 0.8 (elastic net) we used 1000 replicates. Gene importance and variable importance plots were inferred using the variable importance plots (*vip*) package^39^. To determine whether there is a change in gene importance over time, we calculated each gene’s importance for every optimized elastic net binomial regression model with *α* = 0.8, respectively. Then we fitted a linear curve to every gene’s importance trajectory across the four clinical benefit intervals. High positive coefficients (slope) and correlation coefficients (Pearson r) indicated inclusion of a gene in the signature for clinical benefit over subsequent intervals. Heatmap plots were inferred using (*gplots*) and (*RColorBrewer*) packages^40^. For our final prediction Lasso models (at 6 and 24 months with independent training and validation) we also used 1000 replicates to obtain the best model. Specificity, sensitivity, positive predictive value (PPV), negative predictive value (NPV), accuracy, and *p*-value were calculated using the confusion matrix from (*caret*) package, while ROC curves were drawn using the (*ROCR*) package^41,42^. Finally, we used the Youden’s index to calculate the optimal cutoff and separate patients into high and low risk subgroups. Kaplan-Meier curves and log rank test were implemented using (*survival*) and (*survminer*) packages^43,44^. The entire analysis was performed using R 4.1.2.

### Reporting summary

Further information on research design is available in the Nature Research Reporting Summary linked to this article.

## Supplementary information

Supplemental Material

REPORTING SUMMARY

## Data Availability

The data that support the findings of this study have been deposited in NCBI’s Gene Expression Omnibus and are accessible through GEO Series accession number GSE215868. The TCGA-SKCM can be accessed at https://cancergenome.nih.gov. The dataset by Gide et al. used for external validation of our findings can be accessed through the corresponding publication^38^.

## Code availability

For replicating stochasticity, we used “set.seed(14752)”. For the cv.glmnet function our models were developed using type.measure = ”auc”, nfolds = 4, family = binomial, alpha = 1 for lasso regression, otherwise we used alpha Î [0,1) for elastic net or ridge regression and nlambda = 1000. Our best lambda value for our signature model was 0.02069792. The R code that was used for generating our signature model can be provided upon request to corresponding authors or through github (https://github.com/Lsalichos/LBT_code). Our LTB model coefficients are provided in Supplementary Table 2.

## References

[1] Guy GP (2015). Vital signs: melanoma incidence and mortality trends and projections - United States, 1982–2030. Morb. Mortal. Wkly Rep..

[2] Siegel RL (2022). Cancer statistics, 2022. CA Cancer J. Clin..

[3] Siegel RL (2021). Cancer Statistics, 2021. CA Cancer J. Clin..

[4] Larkin J (2019). Five-year survival with combined nivolumab and ipilimumab in advanced melanoma. N. Engl. J. Med..

[5] Hamid O (2019). Five-year survival outcomes for patients with advanced melanoma treated with pembrolizumab in KEYNOTE-001. Ann. Oncol..

[6] Michielin, O. et al. Evolving impact of long-term survival results on metastatic melanoma treatment. *J Immunother. Cancer* **8** (2020). 10.1136/jitc-2020-000948 PMC7549477 33037115

[7] Daud AI (2016). Programmed death-ligand 1 expression and response to the anti-programmed death 1 antibody pembrolizumab in melanoma. J. Clin. Oncol..

[8] Topalian SL (2012). Safety, activity, and immune correlates of anti-PD-1 antibody in cancer. N. Engl. J. Med..

[9] Tumeh PC (2014). PD-1 blockade induces responses by inhibiting adaptive immune resistance. Nature.

[10] Ayers M (2017). IFN-γ-related mRNA profile predicts clinical response to PD-1 blockade. J. Clin. Invest..

[11] Morrison C (2018). Predicting response to checkpoint inhibitors in melanoma beyond PD-L1 and mutational burden. J. Immunother. Cancer.

[12] Conroy JM (2018). Analytical validation of a next-generation sequencing assay to monitor immune responses in solid tumors. J. Mol. Diagn..

[13] Gubin MM (2014). Checkpoint blockade cancer immunotherapy targets tumour-specific mutant antigens. Nature.

[14] Rizvi NA (2015). Cancer immunology. Mutational landscape determines sensitivity to PD-1 blockade in non-small cell lung cancer. Science.

[15] Lu S (2019). Comparison of biomarker modalities for predicting response to PD-1/PD-L1 checkpoint blockade: a systematic review and meta-analysis. JAMA Oncol..

[16] Eisenhauer EA (2009). New response evaluation criteria in solid tumours: revised RECIST guideline (version 1.1). Eur. J. Cancer.

[17] Mulkey, F. et al. Comparison of iRECIST versus RECIST V.1.1 in patients treated with an anti-PD-1 or PD-L1 antibody: pooled FDA analysis. *J. Immunother. Cancer* **8** (2020). 10.1136/jitc-2019-000146 PMC7057528 32107275

[18] Robert C (2018). Durable complete response after discontinuation of pembrolizumab in patients with metastatic melanoma. J. Clin. Oncol..

[19] Ott PA (2019). T-cell-inflamed gene-expression profile, programmed death ligand 1 expression, and tumor mutational burden predict efficacy in patients treated with pembrolizumab across 20 cancers: KEYNOTE-028. J. Clin. Oncol..

[20] Cristescu, R. et al. Pan-tumor genomic biomarkers for PD-1 checkpoint blockade-based immunotherapy. *Science* **362** (2018). 10.1126/science.aar3593 PMC6718162 30309915

[21] Fojo AT, Noonan A (2012). Why RECIST works and why it should stay—counterpoint. Cancer Res..

[22] Betof Warner A (2020). Long-term outcomes and responses to retreatment in patients with melanoma treated with PD-1 blockade. J. Clin. Oncol..

[23] Horn L (2017). Nivolumab Versus Docetaxel in Previously Treated Patients with Advanced Non-Small-Cell Lung Cancer: Two-Year Outcomes from Two Randomized, Open-Label, Phase III Trials (CheckMate 017 and CheckMate 057). J. Clin. Oncol..

[24] Schadendorf D (2015). Pooled analysis of long-term survival data from phase II and phase III trials of ipilimumab in unresectable or metastatic melanoma. J. Clin. Oncol..

[25] Atkins MB (2019). Comparative efficacy of combination immunotherapy and targeted therapy in the treatment of BRAF-mutant advanced melanoma: a matching-adjusted indirect comparison. Immunotherapy.

[26] Wolchok JD (2017). Overall survival with combined nivolumab and ipilimumab in advanced melanoma. N. Engl. J. Med..

[27] Hodi FS (2018). Nivolumab plus ipilimumab or nivolumab alone versus ipilimumab alone in advanced melanoma (CheckMate 067): 4-year outcomes of a multicentre, randomised, phase 3 trial. Lancet Oncol..

[28] Ascierto PA, Long GV (2016). Progression-free survival landmark analysis: a critical endpoint in melanoma clinical trials. Lancet Oncol..

[29] Gibney GT, Weiner LM, Atkins MB (2016). Predictive biomarkers for checkpoint inhibitor-based immunotherapy. Lancet Oncol..

[30] Atefi M (2014). Effects of MAPK and PI3K pathways on PD-L1 expression in melanoma. Clin. Cancer Res..

[31] Pires da Silva I (2019). Distinct molecular profiles and immunotherapy treatment outcomes of V600E and V600K BRAF-mutant melanoma. Clin. Cancer Res..

[32] Vathiotis IA (2021). Models that combine transcriptomic with spatial protein information exceed the predictive value for either single modality. NPJ Precis Oncol..

[33] Guang S (2010). Small regulatory RNAs inhibit RNA polymerase II during the elongation phase of transcription. Nature.

[34] Jiao AL (2019). Human nuclear RNAi-defective 2 (NRDE2) is an essential RNA splicing factor. RNA.

[35] Richard P (2018). NRDE-2, the human homolog of fission yeast Nrl1, prevents DNA damage accumulation in human cells. RNA Biol..

[36] Schipper H, Goh CR, Wang TL (1995). Shifting the cancer paradigm: must we kill to cure?. J. Clin. Oncol..

[37] Dunn GP, Old LJ, Schreiber RD (2004). The three Es of cancer immunoediting. Annu Rev. Immunol..

[38] Gide TN (2019). Distinct immune cell populations define response to anti-PD-1 monotherapy and anti-PD-1/anti-CTLA-4 combined therapy. Cancer Cell.

[39] Greenwell BM, Boehmke BC, Gray B (2020). Variable importance plots-an introduction to the vip package. R. J..

[40] Warnes, M. G. R. et al. Package ‘gplots’. *Various R programming tools for plotting data*, (2016).

[41] Kuhn M (2008). Building predictive models in R using the caret Package. J. Stat. Softw..

[42] Sing T (2005). ROCR: visualizing classifier performance in R. Bioinformatics.

[43] Therneau, T. A package for survival analysis in R. R package version 3.1-12. (eds.) Book A *Package for Survival Analysis in R. R package version*, p. 3.1-12 (2020).

[44] Kassambara, A. et al. Package ‘survminer’. *Drawing Survival Curves using ‘ggplot2’(R package version 03 1)*, (2017).

