# Supplementary material for: Baseline gene expression profiling determines long-term benefit to programmed cell death protein 1 axis blockade
Source: NPJ Precis Oncol. 2022 Dec 15;6:92. doi: 10.1038/s41698-022-00330-3 (PMC9755314; doi:10.1038/s41698-022-00330-3)
Supplement: Supplementary file 1 — Supplemental Material [file 41698_2022_330_MOESM1_ESM.pdf]

Supplementary Table 1. Variability in gene importance as progression-free survival increases.

	Gene importance per time period					
**GENES**	**6**	**12**	**18**	**24**	**Linear Coefficient**	**Correlation Coef**
APOL6	0	0	0	0.05	243.8549852	0.774596669
BAD	0	0	0.39	2	7.032438926	0.866106631
C2	0	0	0	0.3	40.43774145	0.774596669
CCL8	0.01	0	0	0	-1337.566857	-0.774596669
CD274	0	0	0	0.27	44.10806687	0.774596669
COL11A1	0.02	0	0	0	-707.0065004	-0.774596669
COL17A1	0.04	0.04	0.05	0	-215.0166777	-0.641006029
CSF2RB	0	0.31	0	0	-13.00000897	-0.25819889
CXCL14	0	0	0.08	0	48.14809023	0.25819889
CXCL5	0	0	0	0.16	74.34345171	0.774596669
CXCL8	0	0.13	0	0.5	24.74638499	0.747701029
CXCL9	0.16	0.17	0	0	-70.17805493	-0.873020223
DEFB134	0.11	0.27	0.15	0.69	23.02215858	0.788294539
EPCAM	0	0	0.06	0	71.01903524	0.25819889
ERBB2	0.01	0.21	0	0	-21.91339958	-0.298467528
F2RL1	0	0	0.13	0.13	90.13138793	0.879561107
GMIP	0	0.07	0.07	1.15	11.32231589	0.805782418
GPSM3	1.09	0.42	0	0	-13.98485964	-0.92608817
HDAC11	0	0	0.41	0	9.866235064	0.25819889
HLA.DQA2	0	0.08	0	0.36	34.14595715	0.755675994
HLA.F	0	0.04	0	1.25	9.655014115	0.7742974
HMGA1	0	0.11	0	0.71	17.46843941	0.764789876
HMGB1	0.82	0.71	0.51	0.84	-5.55742632	-0.109432729
HSD11B1	0	0.11	0	0.18	55.73236723	0.645915414
IDO1	0.02	0.02	0.12	0.61	23.67548765	0.856416985
IFI35	0.14	0	0	0	-85.52350429	-0.774596669
IKBKG	0	0	0	0.07	164.9908587	0.774596669
IL21R	0	0	0	1.04	11.49965847	0.774596669
IL2RB	0	0	0	0.14	86.73832425	0.774596669
ITGA6	0.38	0.52	0	0.05	-23.40373647	-0.76906888
LAMB3	0	0	0	0.13	95.5620464	0.774596669
LDHB	0	0.42	0	0.04	-7.374081056	-0.195974812
LGALS9	0	0	0	0.6	19.95500461	0.774596669
LYZ	0.05	0	0	0.5	22.73669487	0.719792804
MGMT	0.09	0.37	0.12	0.14	-5.993771141	-0.100304208
MICB	0	0.09	0	0.27	44.75303873	0.733534196
MMP7	0	0	0.12	0.27	57.17001008	0.938841889
NCAM1	0	0	0.2	0	20.09981573	0.25819889
NRDE2	0	0.09	0	0	-46.93148567	-0.25819889
P4HA2	0	0.45	0.18	1.38	10.24733723	0.813086266
PCK2	0.19	0.23	0.4	0.18	12.1445085	0.156942935
PDGFB	0.12	0.51	0	0	-14.74567988	-0.458351459
PDZK1IP1	0.08	0.12	0	0	-103.1539983	-0.797729833
PIK3CA	0	0.27	0	5.11	2.385684604	0.773608955
POLD1	0.62	0.43	0	0.18	-23.47435939	-0.826375786
PSMB10	0.04	0.33	0.51	1.86	8.659488296	0.902695752
PVR	0	0.11	0.66	1.61	9.980472107	0.944592856
RAD51	0.41	0	0	0	-29.48019352	-0.774596669
RB1	0.94	0.54	0	1.92	3.630884409	0.380783038
RNLS	0	0.12	0	0	-34.32352692	-0.25819889
RUNX3	0	0.06	0	0.04	58.80370779	0.228887324
STAT1	0	0	0	0.05	246.5239103	0.774596669
TLR3	0.35	0	0	0	-34.56986178	-0.774596669
TNFRSF11A	0	0.3	0.1	0.32	30.90655542	0.629381378
TNFSF13	0	0.03	0	0	-147.0636589	-0.25819889
TSLP	0	0.01	0	0	-299.0050052	-0.25819889
WDR76	0	0.5	0	0.46	11.44483311	0.409192562
WNT5B	0.25	0.58	0	0	-17.61119724	-0.62695558

Gene	Coefficient	Function	Signaling pathway
ERBB2	0.03154348	Receptor tyrosine kinase; proliferation and survival	Various, including PI3K/Akt and MAPK/ERK
PIK3CA	-0.27556654	Lipid kinase; proliferation and survival	PI3K/Akt
NRDE2	-0.40016433	RNA degradation; DNA damage response	NA
INHBA	0.16432095	Inhibin beta A subunit; tumor suppressor	NA
MMP7	0.01911488	Degradation of the extracellular matrix; invasion and metastasis	Wnt/β catenin, TGF-β
P4HA2	0.32543580	Collagen synthesis	NA
MYD88	0.25137789	Signal transducer; proinflammatory	Innate immune system; IL-1, TLR, NF-κB
DEFB134	0.34902263	Defensin; proinflammatory	Innate immune system
PSMB10	0.14860176	Immunoproteasome; antigen presentation by MHC class I	STAT1/STAT3, IFNγ; NF-κB
HLA.DQA2	0.18764448	Antigen presentation by HLA class II	IFNγ
CXCL8	-0.26940874	Chemokine	IL-8
IDO1	0.29503052	Tryptophan catabolism; suppression of T cell proliferation, induction of Tregs	Constitutive (by PI3K/Akt) and inducible (by IFNγ) expression

Supplementary Table 2. Components of the LTB gene signature. LTB, long-term benefit.

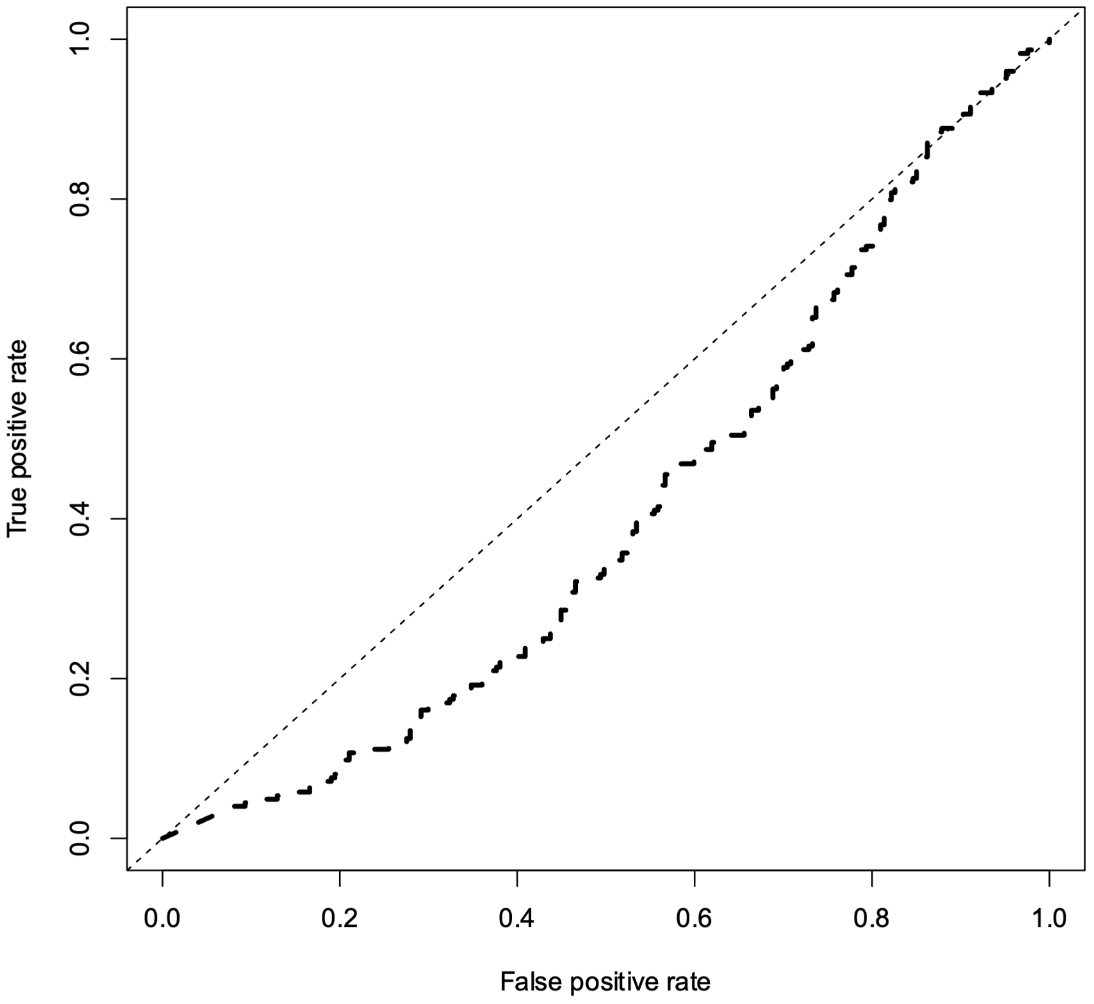

Supplementary Figure 1. Performance of the LTB signature in the TCGA melanoma dataset. LTB, long-term benefit.

Supplementary Figure 2. Performance of the LTB set of 11 out of 12 genes (without HLA.DQA2) in the external validation (Gide et al) dataset. LTB, long-term benefit.

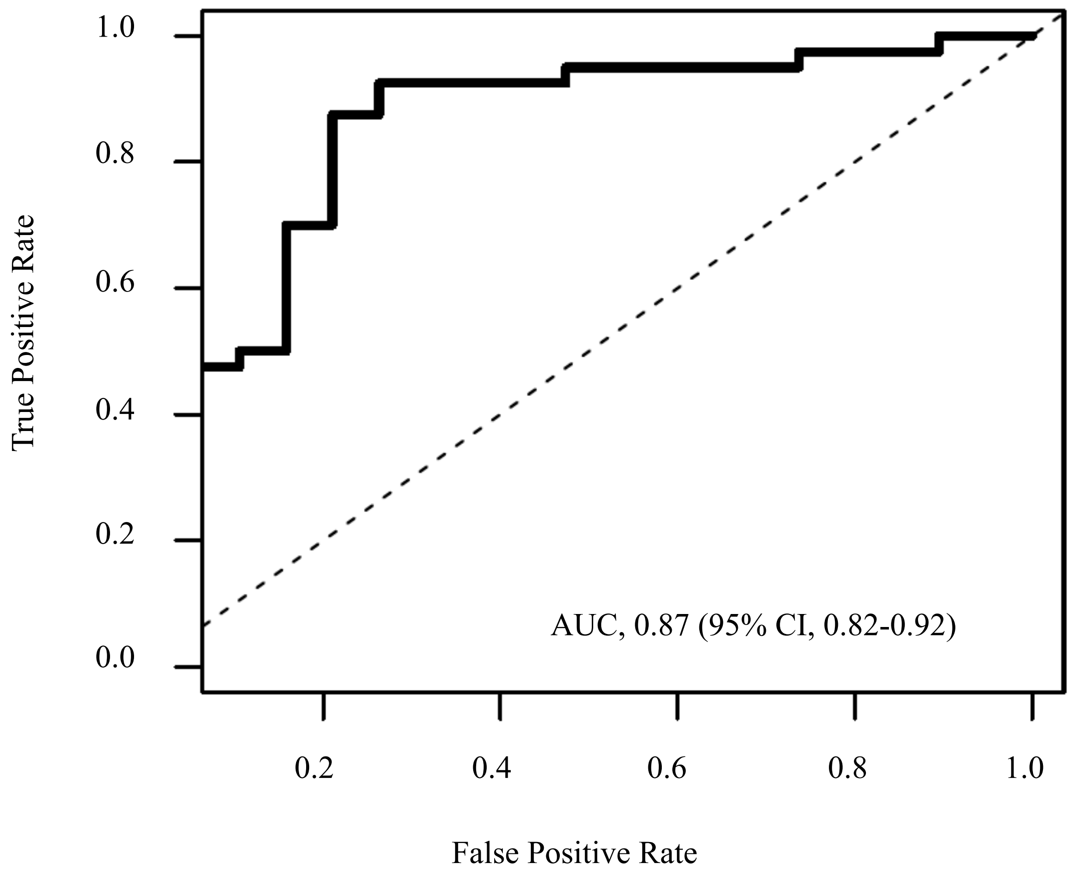

The complete list of genes included in the nCounter PanCancer IO 360™ Panel can be accessed at https://nanostring.com/support-documents/io-360-gene-list/.
