# Supplementary material for: Baseline gene expression profiling determines long-term benefit to programmed cell death protein 1 axis blockade
Source: NPJ Precis Oncol. 2022 Dec 15;6:92. doi: 10.1038/s41698-022-00330-3 (PMC9755314; doi:10.1038/s41698-022-00330-3)
Supplement: Supplementary file 2 — REPORTING SUMMARY [file 41698_2022_330_MOESM2_ESM.pdf]

nature portfolio

Corresponding author(s): Ioannis Vathiotis

Last updated by author(s): Aug 25, 2022

# Reporting Summary

Nature Portfolio wishes to improve the reproducibility of the work that we publish. This form provides structure for consistency and transparency in reporting. For further information on Nature Portfolio policies, see our Editorial Policies and the Editorial Policy Checklist.

## Statistics

For all statistical analyses, confirm that the following items are present in the figure legend, table legend, main text, or Methods section.

| n/a | Confirmed | |
|---|---|---|
| ☐ | ☒ | The exact sample size (*n*) for each experimental group/condition, given as a discrete number and unit of measurement |
| ☐ | ☒ | A statement on whether measurements were taken from distinct samples or whether the same sample was measured repeatedly |
| ☐ | ☒ | The statistical test(s) used AND whether they are one- or two-sided<br>*Only common tests should be described solely by name; describe more complex techniques in the Methods section.* |
| ☐ | ☒ | A description of all covariates tested |
| ☐ | ☒ | A description of any assumptions or corrections, such as tests of normality and adjustment for multiple comparisons |
| ☐ | ☒ | A full description of the statistical parameters including central tendency (e.g. means) or other basic estimates (e.g. regression coefficient) AND variation (e.g. standard deviation) or associated estimates of uncertainty (e.g. confidence intervals) |
| ☐ | ☒ | For null hypothesis testing, the test statistic (e.g. *F*, *t*, *r*) with confidence intervals, effect sizes, degrees of freedom and *P* value noted<br>*Give P values as exact values whenever suitable.* |
| ☐ | ☒ | For Bayesian analysis, information on the choice of priors and Markov chain Monte Carlo settings |
| ☐ | ☒ | For hierarchical and complex designs, identification of the appropriate level for tests and full reporting of outcomes |
| ☐ | ☒ | Estimates of effect sizes (e.g. Cohen's *d*, Pearson's *r*), indicating how they were calculated |

*Our web collection on statistics for biologists contains articles on many of the points above.*

## Software and code

Policy information about availability of computer code

| | |
|---|---|
| Data collection | No software was used. |
| Data analysis | The entire analysis was performed using R 3.6.0. |

For manuscripts utilizing custom algorithms or software that are central to the research but not yet described in published literature, software must be made available to editors and reviewers. We strongly encourage code deposition in a community repository (e.g. GitHub). See the Nature Portfolio guidelines for submitting code & software for further information.

## Data

Policy information about availability of data

All manuscripts must include a data availability statement. This statement should provide the following information, where applicable:

- Accession codes, unique identifiers, or web links for publicly available datasets
- A description of any restrictions on data availability
- For clinical datasets or third party data, please ensure that the statement adheres to our policy

The data that support the findings of this study are available at https://nam12.safelinks.protection.outlook.com/?url=https%3A%2F%2Fdoi.org%2F10.6084%2Fm9.figshare.20598117.v1&data=05%7C01%7Cioannis.vathiotis%40yale.edu%7C78ccd4b27e10438e4d1d08da85e031a1%7Cdd8cbebb21394df8b4114e3e87abeb5c%7C0%7C0%7C637969495195118048%7CUnknown%

7CTWFpbGZsb3d8eyJWIjoiMC4wLjAwMDAiLCJQIjoiV2luMzIiLCJBTiI6Ik1haWwiLCJXVCI6Mn0%3D%7C3000%7C%7C%
7C&sdata=fidTg2Nzi3LQrOfvYYjRYIHVVwB5DjzWhvA3DQ3Up0U%3D&reserved=0.

## Human research participants

Policy information about studies involving human research participants and Sex and Gender in Research.

| | |
|---|---|
| Reporting on sex and gender | Our findings do not apply to one sex or gender. Sex and gender were not considered in study design. Sex was determined based on what was assigned at birth. Our analysis included 60 males and 45 females. No sex- or gender-based analysis was performed as we did not expect to find differential response to immune checkpoint blockade based on either gender or sex. |
| Population characteristics | Median age of study participants was 63 years, ranging from 16 to 88 years. Activating BRAF and NRAS mutations were present in 32 (31%) and 18 (17%), respectively. All patients received PD-1-based immunotherapies in the advanced setting. Fifty-eight patients (55%) received anti-PD-1 monotherapy, including 32 treated with pembrolizumab and 26 treated with nivolumab, and 47 patients (45%) received combination immunotherapy with anti-CTLA-4 plus anti-PD-1 (ipilimumab plus nivolumab). It should be noted that 22 patients (21%) had received ipilimumab monotherapy in a previous line of treatment. |
| Recruitment | The study cohorts are retrospective collections of melanoma patients with available tissue, treated with PD-1-based immunotherapies in the advanced setting from 2011 to 2017 (discovery cohort) and from 2017 to 2020 (validation cohort) at Yale Cancer Center (New Haven, CT). Patients with uveal melanoma were excluded. Potential bias stems from the retrospective study design. |
| Ethics oversight | All patients provided written informed consent or waiver of consent. The study was approved by the Yale Human Investigation Committee protocol #9505008219 and conducted in accordance with the Declaration of Helsinki. |

Note that full information on the approval of the study protocol must also be provided in the manuscript.

# Field-specific reporting

Please select the one below that is the best fit for your research. If you are not sure, read the appropriate sections before making your selection.

☒ Life sciences ☐ Behavioural & social sciences ☐ Ecological, evolutionary & environmental sciences

For a reference copy of the document with all sections, see nature.com/documents/nr-reporting-summary-flat.pdf

# Life sciences study design

All studies must disclose on these points even when the disclosure is negative.

| | |
|---|---|
| Sample size | The study cohorts are retrospective collections of melanoma patients with available tissue, treated with PD-1-based immunotherapies in the advanced setting from 2011 to 2017 (discovery cohort) and from 2017 to 2020 (validation cohort) at Yale Cancer Center (New Haven, CT). Sample size was deemed sufficient due to the presence of a training set and a validation set with more than 45 patients each. |
| Data exclusions | Patients with uveal melanoma were excluded, because this represents a unique disease entity with different prognosis than skin/cutaneous melanoma. Exclusion criterion was pre-established. |
| Replication | We validated our 12-gene signature in a validation set with 46 patients from the same institution. We also validated the predictive value of our signature genes in a publicly available dataset (Gide et al). All validation efforts were successful. |
| Randomization | The study was not randomized. Study design is retrospective. |
| Blinding | The investigators were not blinded to group allocation. Study design is retrospective. |

# Reporting for specific materials, systems and methods

We require information from authors about some types of materials, experimental systems and methods used in many studies. Here, indicate whether each material, system or method listed is relevant to your study. If you are not sure if a list item applies to your research, read the appropriate section before selecting a response.

## Materials & experimental systems

| n/a | Involved in the study |
|---|---|
| ☒ | ☐ Antibodies |
| ☒ | ☐ Eukaryotic cell lines |
| ☒ | ☐ Palaeontology and archaeology |
| ☒ | ☐ Animals and other organisms |
| ☐ | ☒ Clinical data |
| ☒ | ☐ Dual use research of concern |

## Methods

| n/a | Involved in the study |
|---|---|
| ☒ | ☐ ChIP-seq |
| ☒ | ☐ Flow cytometry |
| ☒ | ☐ MRI-based neuroimaging |

# Clinical data

Policy information about clinical studies

All manuscripts should comply with the ICMJE guidelines for publication of clinical research and a completed CONSORT checklist must be included with all submissions.

Clinical trial registration

No clinical trial registration. This is a retrospective study.

Study protocol

Not available. This is a retrospective study.

Data collection

The study cohorts are retrospective collections of melanoma patients with available tissue, treated with PD-1-based immunotherapies in the advanced setting from 2011 to 2017 (discovery cohort) and from 2017 to 2020 (validation cohort) at Yale Cancer Center (New Haven, CT). Pretreatment formalin-fixed, paraffin-embedded (FFPE) specimens from Yale Pathology archives were reviewed by a board-certified pathologist. Clinicopathological data were collected from clinical records and pathology reports; the data cutoff date was September 1, 2020.

Outcomes

RECIST 1.1 were used to classify best overall response as complete response (CR), partial response (PR), stable disease (SD), or progressive disease (PD), and to determine the objective response rate (ORR). PFS was utilized to generate successive, 6-month clinical benefit intervals; short-term benefit (STB) was defined for patients who were alive and free of disease progression within 6 months from treatment initiation and long-term benefit (LTB) for those who were alive and free of disease progression within 24 months from treatment initiation. Patients whose follow-up was shorter than the prespecified intervals were excluded from the analysis.
